# High-Risk Suicide Locations in Australia

**DOI:** 10.1001/jamanetworkopen.2024.17770

**Published:** 2024-06-20

**Authors:** Lay San Too, Sangsoo Shin, Suzanne Mavoa, Phillip Cheuk Fung Law, Angela Clapperton, Leo Roberts, Ella Arensman, Matthew J. Spittal, Jane Pirkis

**Affiliations:** 1 Centre for Mental Health and Community Wellbeing, Melbourne School of Population and Global Health, The University of Melbourne, Parkville, Victoria, Australia; 2 Environment Protection Authority Victoria, Carlton, Victoria, Australia; 3 School of Public Health, College of Medicine and Health, University College Cork, Cork, Ireland; 4 National Suicide Research Foundation, University College Cork, Cork, Ireland; 5 Australian Institute for Suicide Research and Prevention, School of Applied Psychology, Griffith University, Brisbane, Queensland, Australia

## Abstract

**Importance:**

Although several interventions have been shown to be effective in preventing suicide at high-risk locations, the potential for these interventions to be deployed is limited by a lack of knowledge about where high-risk locations are and the factors associated with choosing these locations.

**Objective:**

To identify high-risk suicide locations in Australia and the factors associated with choosing these locations.

**Design, Setting, and Participants:**

This case-control study included data on individuals who died by suicide in Australia between January 2001 and December 2017, obtained from the National Coronial Information System. Data analysis was conducted from February to December 2021.

**Exposures:**

Sociodemographic, residential, incident time, and incident location variables.

**Main Outcomes and Measures:**

The scan statistic was used to detect spatial clusters of suicides in public locations. Suicide locations within significant clusters with at least 0.5 suicides per year were defined as high-risk locations. Multivariable logistic regression analyses were performed to examine the factors associated with choosing a high-risk location.

**Results:**

Over the study period, 10 701 suicides took place in public places. The individuals who died of suicide in public places included 8602 (80.4%) male individuals, and most were aged 25 to 49 years (5825 [54.5%]). A total of 17 high-risk suicide locations in Australia were detected. These involved 495 suicides, which accounted for 4.6% of suicides in public locations. For suicides at high-risk locations, 82.2% (407 of 495) occurred at cliffs and bridges. Being female (adjusted odds ratio [aOR], 1.73; 95% CI, 1.41-2.13), employed (aOR, 1.57; 95% CI, 1.20-2.04), never married (aOR, 1.64; 95% CI, 1.26-2.13), and from a major city (aOR, 3.94; 95% CI, 2.94-5.28) were associated with the choice of a high- over low-risk suicide location. High-risk locations tended to be in major cities.

**Conclusions and Relevance:**

This case-control study found 17 high-risk suicide locations in Australia and the factors associated with the choice of these locations. Actions should be taken to prevent suicide at these locations where possible.

## Key Points

**Question:**

Where are high-risk suicide locations in Australia, and what factors are associated with the choice of suicide location?

**Findings:**

This case-control study with 10 701 suicide deaths detected 17 high-risk suicide locations in Australia; for suicides at these locations, 82.2% occurred at cliffs and bridges. Being female, employed, never married, and from a major city were associated with choosing a high- over low-risk suicide location.

**Meaning:**

The comprehensive list of high-risk suicide locations and the factors associated with the choice of high-risk locations can be used to design and implement suicide prevention interventions at the locations.

## Teaser

This cross-sectional study examines what locations in Australia are high risk for suicide and what sociodemographic and residential characteristics are associated with the choice of a high-risk suicide location.

## Introduction

Suicide is an increasingly recognized public health priority that warrants a sustained multipronged approach.^1^ There were 3166 suicides in Australia in 2021, equating to 9 suicides per day.^2^ Every suicide is a tragedy that has a devastating and long-lasting adverse impact on families, friends, communities, and society. Suicide also has major economic impacts, costing more than AU $1.6 billion per year (to convert Australian dollars to US dollars, multiply by 0.67).^3^

Certain sites have been identified as so-called suicide hotspots, which are defined as specific, accessible, and usually public locations in which suicides are frequent.^4^ There is no universal agreement, but because of criticisms about the use of the term suicide hotspots,^5^ we refer to these places as high-risk suicide locations. Media reports and word-of-mouth on suicide sites can perpetuate the reputations of these sites as high-risk locations and create contagion effect (ie, individuals select these locations because they know others have gone there to attempt suicide).^4^ Some high-risk suicide locations offer the means of suicide (eg, bridges and cliffs provide an opportunity for jumping).^6,7^ Other high-risk suicide locations offer seclusion, which may allow suicide attempts to proceed uninterrupted (eg, bushlands and forests).^8^

Preventing suicides at high-risk locations is crucial because these locations can be self-perpetuating and because of the adverse impacts suicides at these locations have for those who live near, work near, or witness them. The evidence to date is that some interventions are useful for reducing suicides at high-risk locations. A meta-analysis^9^ concluded that restricting access to means (eg, installing physical barriers) reduced the number of suicides at high-risk locations by 91%. Other strategies, such as encouraging help-seeking (eg, signs displaying a helpline contact number) and increasing the likelihood of third-party intervention (eg, installing closed-circuit television cameras and implementing police patrols) were also effective, reducing the number of suicides at high-risk locations by 51% and by 47%, respectively.^9^

Despite the potential to prevent suicides by intervening at high-risk locations, no studies have systematically identified these locations in Australia. Knowing the locations where suicides frequently occur would enable authorities to focus interventions, resulting in effective suicide prevention. Therefore, the first aim of this study was to systematically identify high-risk suicide locations in Australia. Following this, the second aim was to examine the factors associated with the choice of a high- over low-risk suicide location. Knowing the associated factors would add to existing knowledge and may help in designing third-party interventions to prevent suicide at high-risk locations.

## Methods

We first detected high-risk suicide locations. Then we used a case-control design to examine the factors associated with the choice of a high- over low-risk location.

### Suicide Data

Data on deaths classified as intentional self-harm (considered suicides) in Australia were obtained from the National Coronial Information System (NCIS). The NCIS is an internet-based data storage and retrieval system of Australian and New Zealand coronial records.^10^ It contains data on deaths reported to an Australian coroner from July 2000 (except Queensland, where data are available from January 2001) and deaths reported to a New Zealand coroner from July 2007. For each record in the NCIS, there is coded data, and most have associated full-text reports. The coded data contains information on the classification of the person’s death, the details of their death, and their sociodemographic characteristics. The text reports contain a police summary of circumstances, an autopsy report, a toxicology report, and the coroner’s findings. Ethical approval for this study was granted by the Health Sciences Human Ethics Committee (the University of Melbourne) and the Victorian Justice Human Research Ethics Committee. We used information from deceased persons who were unable to provide informed consent, but we had an access agreement with the NCIS to ensure that our study was conducted to serve the public interest. This study follows the Strengthening the Reporting of Observational Studies In Epidemiology (STROBE) reporting guideline.

### Inclusion and Exclusion Criteria

We included suicides that occurred between January 2001 and December 2017, the most recent year for which complete data were available from the NCIS at the time of data extraction. For suicide cases for which the specific incident date was not available and a date range (eg, between November 2006 and March 2007) was provided, we included cases if their date range overlapped with the study period. For example, a suicide that occurred between November 2017 and January 2018 and a suicide that occurred between December 2000 and January 2001 were included. We classified the location of suicides into 2 groups: public locations (ie, those open to the public, such as bridges, cliffs, public parks, car parks, rail tracks or stations) and nonpublic locations (ie, those with restricted access and/or owned by the person who died, such as homes or residential institutions) (eTable in Supplement 1). This classification was based on the coded location type extracted from the NCIS (cases with unspecific location type were manually coded according to the information available in the text reports). For suicides that occurred in hospitals, we classified those that took place in the hospital car park as occurring in public locations and those that took place on an inpatient ward as occurring in nonpublic locations. We included only suicide cases in public locations in our analyses.

### Variables of Interest

For each suicide, we extracted coded information on *International Statistical Classification of Diseases and Related Health Problems, Tenth Revision *(*ICD-10*) code, cause of death, primary mechanism of suicide, object used in suicide, sex, age, employment status, marital status, incident date, and location type as well as the addresses of incident and residential locations (including their geographical coordinates). Data on race and ethnicity are not always collected, coded, and stored in the NCIS, so they were not included in this study. Suicide method was coded based on all the information provided in the *ICD-10* code, cause of death, mechanism of suicide, and object used in suicide. Remoteness of residence and incident locations were coded using the relevant geographical coordinates. Incident day and season were coded using incident date.

### Statistical Analysis

We developed a novel 2-step procedure to identify high-risk suicide locations in Australia. In our first step, we used the scan statistic to identify statistical suicide clusters based on the information on suicide locations. The scan statistic is a statistical method for detecting nonrandom clusters in time and/or space.^11^ In other words, it can be used to systematically identify locations where there is an unusually high number of deaths with respect to chance.^12^ This method has been used to detect statistical suicide clusters.^13,14,15^ We focused on statistical clusters of suicides occurring in space only (and not time) on the grounds that suicides at specific high-risk locations might take place over several years. To do this, we identified spatial clusters of high relative risk for suicide using the Poisson discrete scan statistic in SaTScan version 9.7 (SaTScan), with mesh blocks as the spatial unit of analysis. Mesh blocks are the smallest geographical area in the Australian Statistical Geography Standard defined by the Australian Bureau of Statistics (ABS).^16^ To run the analysis in SaTScan, information on cases, population estimates, and area coordinates were required to be set in a particular format. We therefore assigned the mesh block for each case based on the geographical coordinates of suicide locations using geographic information systems (GIS) software (ArcGIS version 10.8.1 [ESRI]). We obtained population estimates for each mesh block from the ABS census data in 2011. We used ArcGIS software to compute the geographical coordinates of the centroid of each mesh block based on the 2011 ABS digital mesh block boundaries. After importing the required data into SaTScan, we prespecified a 2-km radius as the maximum size of the spatial scan window and selected circular as the shape of the window. These settings were chosen based on our pilot work identifying the optimal settings for detecting well-known high-risk suicide locations. The selection of a 2-km radius was also because the longest bridge in Australia is 3.2 km in length, which meant this spatial parameter would be able to detect any high-risk bridges in Australia if such clusters existed. The statistical likelihood of each potential cluster was assessed using Monte Carlo simulation.^11^ Clusters were classified as statistically significant if their *P* value was less than .05.

In our second step, after identifying statistical spatial clusters of suicides at public locations, we retrieved information on the locations of suicides (eg, location name and address) that occurred within the clusters and calculated the suicide number for each location. We adapted the definition of high-risk suicide locations from a recent Swiss study,^17^ where these locations were defined as those sites with at least 0.5 suicides per year over a 10-year period. Using 17 years of data, we defined and classified locations with at least 9 suicides as a high-risk suicide location. After identifying high-risk suicide locations, we searched their name in our dataset to find any suicides that were not within but located near the relevant statistical cluster to accurately estimate the number of suicides for each location. Cases without a mesh block were not included in our scan analysis (481 [4.5%]). However, to avoid missing potential high-risk suicide locations, for these suicide cases, we manually went through their suicide location information and classified high-risk suicide locations using the same definition as described previously.

Subsequently, we classified suicides at public places that were not at high-risk locations as suicides at low-risk locations. We used a case-control design and performed logistic regression analysis (in Stata version 15.1 [StataCorp]) to examine the factors associated with choosing high-risk locations. The outcome was a binary variable representing whether the suicide was at high- or low-risk location. The exposures were sociodemographic, residential, incident time, and incident location variables. At first, we performed univariate analyses to estimate crude, unadjusted odds ratios (ORs) for suicides at high- and low-risk locations associated with the aforementioned variables. We then performed multivariable analyses to estimate their adjusted ORs (aORs). We also conducted sensitivity analyses (excluding cases with missing information on included variables) to estimate the aORs.

## Results

### High-Risk Suicide Locations

A total of 10 701 suicides occurred in public places between 2001 and 2017 in Australia, accounting for 25.0% of all suicides (n = 42 712). The individuals who died by suicide were predominantly male individuals (8602 [80.4%]), and the largest age group was 25 to 49 years (5825 [54.5%]). In the first step, we detected 238 significant statistical suicide clusters including 1814 suicides. In the second step, we identified 17 locations that met our definition of high-risk suicide locations (ie, at least 9 suicides) among the detected statistical clusters (Table 1). Nine of these locations were in New South Wales (52.9%), 3 in Victoria (17.6%), 3 in Queensland (17.6%), 1 in Western Australia (5.9%), and 1 in Tasmania (5.9%). A total of 495 suicides occurred at high-risk locations (4.6% of suicides in public places and 1.2% of all suicides). At 10 high-risk suicide locations, 2 or more suicide methods were used; while at 7 locations, only 1 method was used. For the specific type of suicide location, 211 (42.6%) at high-risk locations occurred at a cliff and 196 (39.6%) at a bridge. The remaining suicides occurred in a public park (39 [7.9%]), on the railway (21 [4.2%]), in a parking area (18 [3.6%]), and in a shopping center, remote or undeveloped place, roadway, or footpath (10 [2.0%]).

**Table 1.** Information on High-Risk Suicide Locations

No.	State or territory	Location type	Suicide method^a^	Suicides, No. (%) (n = 495)
1	New South Wales	Cliff	>90 Jumping from a height ≥5 Drowning <5 Colliding into a motor vehicle	106 (21.4)^b^
2	Victoria	Bridge	77 Jumping from a height 14 Drowning	91 (18.4)
3	Queensland	Bridge	44 Jumping from a height 14 Drowning	58 (11.7)^b^
4	New South Wales	Cliff, footpath	>30 Jumping from a height <5 Hanging or asphyxia	35 (7.1)^b^
5	New South Wales	Cliff, remote or undeveloped area, parking area	>20 Jumping from a height <5 Hanging or asphyxia <5 Poisoning <5 Motor vehicle exhaust gas <5 Colliding into a motor vehicle <5 Unknown or unspecified	30 (6.1)^c^
6	Tasmania	Bridge	18 Drowning 9 Jumping from a height	27 (5.5)^b^
7	New South Wales	Public park, parking area, roadway	11 Hanging or asphyxia <5 Motor vehicle exhaust gas <5 Jumping from a height <5 Poisoning by drugs or substances <5 Colliding into a motor vehicle <5 Others	22 (4.4)^b^^,^^c^
8	Western Australia	Public park	8 Jumping from a height 8 Hanging or asphyxia <5 Poisoning by drugs or substances <5 Motor vehicle exhaust gas <5 Cutting, piercing, or stabbing <5 Unknown or unspecified	21 (4.2)
9	Victoria	Railway (including railway station)	21 Jumping in front of a train	21 (4.2)^b^^,^^c^
10	New South Wales	Cliff	13 Jumping from a height	13 (2.6)
11	Queensland	Cliff	13 Jumping from a height	13 (2.6)
12	Victoria	Bridge	11 Jumping from a height	11 (2.2)^b^
13	New South Wales	Cliff	10 Jumping from a height	10 (2.0)
14	New South Wales	Shopping center, parking area	≥5 Jumping from a height <5 Hanging or asphyxia	10 (2.0)
15	Queensland	Parking area	9 Jumping from a height	9 (1.8)
16	New South Wales	Cliff	9 Jumping from a height	9 (1.8)
17	New South Wales	Bridge	≥5 Jumping from a height <5 Drowning	9 (1.8)

^a^ To ensure no individual can be identified, some high-risk suicide locations are reported with the categories of less than 5 suicides (<5) and/or 5 or more suicides (≥5).

^b^ Some of these suicides were found through text search because they were either not assigned a mesh block or not within but near the cluster.

^c^ Two clusters were detected in these locations.

### Characteristics of Suicides at Low- and High-Risk Locations

Table 2 shows the characteristics of suicides at low- and high-risk suicide locations. A greater proportion of females died at high- vs low-risk suicide locations (143 of 495 [28.9%] vs 1949 of 10 199 [19.1%]). Over three-quarters of individuals younger than 49 years old chose high-risk locations (377 [76.2%]), while a lower proportion in the same age groups chose low-risk locations (7210 [70.7%]). For those who died by suicide at a high-risk location, 83 (16.8%) were unemployed, and 166 (33.5%) were never married. For those who died at a low-risk location, 2324 (22.8%) were unemployed, and 3058 (30.0%) were never married. Most individuals who died at high-risk locations were from major cities (428 [86.5%]). A lower proportion was observed for those who died at low-risk locations (6407 [62.8%]).

**Table 2.** Characteristics of Suicides at High- and Low-Risk Suicide Locations

Variable	Individuals who died by suicide, No. (%)			*P* value, χ^2^ test
	Total suicides (N = 10 694)^a^	Suicides at high-risk locations (n = 495)	Suicides at low-risk locations (n = 10 199)	
Sociodemographic				
Sex				
Male	8602 (80.44)	352 (71.11)	8250 (80.89)	<.001
Female	2092 (19.56)	143 (28.89)	1949 (19.11)	
Age group, y				
≤24	1762 (16.48)	70 (14.14)	1692 (16.59)	.002
25-49	5825 (54.47)	307 (62.02)	5518 (54.10)	
≥50	3107 (29.05)	118 (23.84)	2989 (29.31)	
Employment status				
Employed	4230 (39.55)	210 (42.42)	4020 (39.42)	<.001
Unemployed	2407 (22.51)	83 (16.77)	2324 (22.79)	
Not in the labor force	2152 (20.12)	87 (17.58)	2065 (20.25)	
Unknown or other^b^	1905 (17.81)	115 (23.23)	1790 (17.55)	
Marital status				
Married, including de facto	3441 (32.18)	128 (25.86)	3313 (32.48)	<.001
Never married	3224 (30.15)	166 (33.54)	3058 (29.98)	
Divorced, separated, or widowed	1969 (18.41)	78 (15.76)	1891 (18.54)	
Unknown	2060 (19.26)	123 (24.85)	1937 (18.99)	
Residential area				
Remoteness of residence				
Major cities	6835 (63.91)	428 (86.46)	6407 (62.82)	<.001
Regional or remote	3359 (31.41)	52 (10.51)	3307 (32.42)	
Unknown	500 (4.68)	15 (3.03)	485 (4.76)	
Incident time and location variables				
Incident day				
Weekday	7493 (70.07)	360 (72.73)	7133 (69.94)	.28
Weekend	2758 (25.79)	120 (24.24)	2638 (25.87)	
Unknown	443 (4.14)	15 (3.03)	428 (4.20)	
Incident season				
Summer (Dec-Feb)	2624 (24.54)	120 (24.24)	2504 (24.55)	.78
Autumn (Mar-May)	2535 (23.70)	124 (25.05)	2411 (23.64)	
Winter (Jun-Aug)	2434 (22.76)	113 (22.83)	2321 (22.76)	
Spring (Sep-Nov)	2668 (24.95)	123 (24.85)	2545 (24.95)	
Unknown	433 (4.05)	15 (3.03)	418 (4.10)	
Remoteness of suicide location				
Major cities	5835 (54.56)	350 (70.71)	5485 (53.78)	<.001
Regional or remote	4378 (40.94)	72 (14.55)	4306 (42.22)	
Unknown^c^	481 (4.50)	73 (14.75)	408 (4.00)	

^a^ Seven cases were removed from the analysis because information on their age was not available.

^b^ This category included 28 cases with other employment status, such as prisoner.

^c^ A larger proportion of unknown remoteness of suicide location was observed in suicides at high-risk locations than those in low-risk locations. This was likely due to the former occurring more often at a bridge or cliff near or above water, and water areas were not assigned remoteness information using Australian Statistical Geography Standard Remoteness Structure that was derived based on road distance.

Suicides at high- and low-risk locations occurred most frequently on a weekday (360 [72.7%] and 7133 [69.9%], respectively). Suicides at both location types occurred quite evenly throughout all seasons. Many suicides at high-risk locations were in major cities (350 [70.7%]), and only about half of suicides at low-risk locations were in major cities (5485 [53.8%]).

### Results From the Multivariable Models

Based on model 1 from the multivariable analysis (Table 3), sex, age, employment status, and marital status were significant factors for choosing a high- over low-risk location. These variables (except age) remained significant after residential remoteness was introduced into the multivariable model 2. In that, being female was significantly associated with the choice of a high- over low-risk suicide location after adjustment for the other variables in the model (aOR, 1.73; 95% CI, 1.41-2.13). Being employed was also a significant factor for choosing a high- over low-risk location (aOR, 1.57; 95% CI, 1.20-2.04). In terms of marital status, those who were never married were more likely to choose a high- over low-risk location compared with those who were married (aOR, 1.64; 95% CI, 1.26-2.13). Those who lived in major cities were also more likely to choose a high- over low-risk location compared with those who lived in regional or remote areas (aOR, 3.94; 95% CI, 2.94-5.28).

**Table 3.** Sociodemographic and Residential Factors Associated With Suicides at High-Risk Locations

Variable	Univariate models, cOR (95% CI)	aOR (95% CI)	
		Multivariable model 1	Multivariable model 2
Sociodemographic			
Sex			
Male	1 [Reference]	1 [Reference]	1 [Reference]
Female	1.72 (1.41-2.10)	1.82 (1.48-2.23)	1.73 (1.41-2.13)
Age group, y			
≤24	1.05 (0.78-1.42)	0.80 (0.57-1.12)	0.82 (0.58-1.14)
25-49	1.41 (1.13-1.75)	1.28 (1.01-1.61)	1.24 (0.98-1.57)
≥50	1 [Reference]	1 [Reference]	1 [Reference]
Employment status			
Employed	1.46 (1.13-1.90)	1.64 (1.26-2.13)	1.57 (1.20-2.04)
Unemployed	1 [Reference]	1 [Reference]	1 [Reference]
Not in the labor force	1.18 (0.87-1.60)	1.31 (0.95-1.81)	1.24 (0.90-1.72)
Unknown or other^a^	1.80 (1.35-2.40)	1.77 (1.31-2.38)	1.56 (1.16-2.11)
Marital status			
Married, including de facto	1 [Reference]	1 [Reference]	1 [Reference]
Never married	1.41 (1.11-1.78)	1.68 (1.30-2.18)	1.64 (1.26-2.13)
Divorced, separated, or widowed	1.07 (0.80-1.42)	1.09 (0.82-1.45)	1.11 (0.83-1.48)
Unknown	1.64 (1.28-2.12)	1.63 (1.25-2.12)	1.52 (1.17-1.99)
Residential area variable			
Remoteness of residence			
Major cities	4.25 (3.18-5.68)	NA	3.94 (2.94-5.28)
Regional or remote	1 [Reference]	NA	1 [Reference]
Unknown	1.97 (1.10-3.52)	NA	1.85 (1.03-3.33)

Abbreviations: aOR, adjusted odds ratio; cOR, crude odds ratio; NA, not applicable.

^a^ This category included 28 cases with other employment status, such as prisoner.

As shown in Table 4, incident day and season were not significantly associated with the choice of high-risk location. In both model 3 and model 4, suicides at high-risk locations more likely occurred in major cities than in regional or remote areas (model 3: aOR, 3.80; 95% CI, 2.94-4.91; model 4: aOR, 3.80; 95% CI, 2.94-4.92). After excluding cases with missing information on included variables, our sensitivity analysis showed that all results remained the same, except for age group, which was not significant in the initial multivariable model.

**Table 4.** Time and Location Factors Associated With Suicides at High-Risk Locations

Variable	Univariate models, cOR (95% CI)	aOR (95% CI)	
		Multivariable model 3	Multivariable model 4
Incident time and location variable			
Incident day			
Weekday	1 [Reference]	1 [Reference]	NA
Weekend	0.90 (0.73-1.11)	0.92 (0.74-1.13)	NA
Unknown	0.69 (0.41-1.17)	0.83 (0.49-1.41)	NA
Incident season			
Summer (Dec-Feb)	1 [Reference]	NA	1 [Reference]
Autumn (Mar-May)	1.07 (0.83-1.39)	NA	1.06 (0.81-1.37)
Winter (Jun-Aug)	1.02 (0.78-1.32)	NA	1.03 (0.79-1.34)
Spring (Sep-Nov)	1.01 (0.78-1.30)	NA	1.02 (0.78-1.32)
Unknown	0.75 (0.43-1.29)	NA	0.88 (0.51-1.53)
Remoteness of suicide location			
Major cities	3.82 (2.95-4.93)	3.80 (2.94-4.91)	3.80 (2.94-4.92)
Regional or remote	1 [Reference]	1 [Reference]	1 [Reference]
Unknown	10.70 (7.61-15.05)	10.66 (7.58-14.99)	10.66 (7.57-14.99)

Abbreviations: aOR, adjusted odds ratio; cOR, crude odds ratio; NA, not applicable.

## Discussion

This study shows that suicides occurring at high-risk locations accounted for 4.6% of suicides in public places. Preventing these suicides is important because of the impact these deaths have on those who may witness them, and because these deaths may be associated with further suicides. There are also particular types of intervention—notably restricting access to means but also encouraging help-seeking and increasing the likelihood of intervention by a third party—that may be more readily implemented to prevent these suicides than suicides that occur in nonpublic locations, such as home.^9,18^

This study also found that cliffs and bridges are the top 2 types of high-risk locations. A recent economic analysis indicated that physical barriers were a cost-effective suicide prevention intervention at bridge sites (but less on cliff sites), which could save an estimated US $145 million in preventing suicide over 5 years and US $270 million over 10 years.^19^ This means preventing suicide at these sites (5 bridges) should be prioritized. Barriers might not prevent all suicides at these sites, but they could prevent the majority that involve jumping from a height. This is supported by the findings of a recent study that showed no jumping suicides at the West Gate Bridge and a decrease in jumping suicides in Victoria (Australia) after installation of the safety barrier.^20^

We found that being female, employed, and never married were significantly associated with the choice of a high- over low-risk suicide location. These characteristics are not common sociodemographic characteristics for all suicides.^21^ The reasons for this profile are unclear. It is possible that an individual’s choice of location may be affected by their knowledge of someone else who has died at the site. Previous research showed that young female individuals were the most vulnerable group for copycat suicide.^22^ Alternatively, high-risk suicide locations may hold a special meaning to individuals who seek them out or the belief that this location provides a minimal chance of being interrupted by a third party.

Our findings show that those who lived in major cities were more likely to choose a high-risk location and high-risk locations tended to be in major cities. However, based on these findings, we could not conclude that access to suicide location is the factor that contributes to the choice of location. Future research could examine the association by including the travel distance between usual residence and suicide location in the analysis.

To our knowledge, our study is the first to identify high-risk suicide locations at all public sites at a national level. We used 17 years of data and a rigorous 2-step approach to systematically identify these locations in Australia. Previous studies primarily relied on suicide counts to define high-risk locations and/or detect these locations in a specific public setting.^17,23,24^ Our approach first adjusts for population number to identify statistical suicide clusters and then detects high-risk suicide locations within the clusters based on the number of suicides. This approach enables detecting high-risk suicide locations within significant clusters due to an increased risk of suicide at the locations (possibly caused by contagion effect or other reasons) but not the difference in geographical population distribution. Our approach has also detected well-known high-risk suicide locations in the country. Importantly, the findings of high-risk locations are directly translatable and could be used to inform relevant authorities as to where to prioritize suicide prevention interventions. Our study is also the first of which we are aware to show the factors associated with choosing high-risk suicide locations.

### Limitations

This study has limitations. First, underreporting of suicides is possible because (1) coronial investigations of cases that occurred within our study period may still have been open at the time of data extraction and (2) suicides may be misclassified as being deaths with undetermined intent or unintentional causes.^2^ Second, given that a high-risk suicide location is defined by the relatively small number of suicides, underreporting of suicides can miss some locations where suicide frequently occur. Third, some geographic coordinates did not match the name of the reported suicide locations. In this case, we obtained their geographic coordinates using Google Maps based on the name of suicide locations and included these coordinates in our analysis. Fourth, suicide preventive interventions have been deployed in some high-risk locations identified from our study, and this was not considered in our analysis.

## Conclusions

In summary, the current study found 17 high-risk suicide locations in Australia where prevention interventions should be targeted. Given that there is good evidence for the effectiveness of various interventions in preventing suicide at high-risk locations, we suggest that preventive actions should be taken at the identified locations where feasible or if they have not already been done.

## Notes

Supplement 1. **eTable.** Classification of Suicide Location Type

Supplement 2.
Data Sharing Statement
