# Supplementary material for: High-Risk Suicide Locations in Australia
Source: JAMA Netw Open. 2024 Jun 20;7(6):e2417770. doi: 10.1001/jamanetworkopen.2024.17770 (PMC11190791; doi:10.1001/jamanetworkopen.2024.17770)
Supplement: Supplement 1. — eTable. Classification of Suicide Location Type [file jamanetwopen-e2417770-s001.pdf]

Supplemental Online Content

Too LS, Shin S, Mavoa S, et al. High-risk suicide locations in Australia. *JAMA Netw Open.* 2024;7(6):e2417770. doi:10.1001/jamanetworkopen.2024.17770

eTable. Classification of Suicide Location Type

This supplemental material has been provided by the authors to give readers additional information about their work.

© 2024 Too LS et al. *JAMA Network Open.*

eTable. Classification of Suicide Location Type

Broad location type	Specific location type	Public place
Home	House	No
	Flat, apartment, terrace house	No
	Farmhouse	No
	Residential caravan, mobile home, houseboat, motor home	No
	Hut, shanty, humpy	No
	Boarding house, hotel, backpackers hostel	No
	Granny flat, bungalow	No
	Indigenous community	No
	Home of religious person	No
	Yacht, boat	No
	Workplace/industry accommodation	No
	Demountable, portable home	No
	Converted dwelling	No
	Other specified type of home	No
	Unspecified type of home	No
Countryside	Area of still water	Yes
	Stream of water	Yes
	Large area of water	Yes
	Marsh, swamp	Yes
	Beach, shore, bank of a body of water	Yes
	Forest, bushland, national park	Yes
	Desert, outback	Yes
	Remote or undeveloped place	Yes
	Base of precipice, cliff	Yes
	Drain, channel	Yes
	Dry creek or riverbed	Yes
	Lookout, viewpoint	Yes
	Glacier	Yes
	Hot spring, thermal pool	Yes
	Other specified countryside	Yes
	Unspecified countryside	Yes
Road transport area	Roadway	Yes
	Footpath	Yes
	Cycleway, bike path	Yes
	Highway, freeway	Yes
	Rest area, parking bay	Yes
	Bush track, dirt road	Yes
	Other specified public highway, road	Yes
	Unspecified public highway, road	Yes
Non-road transport area	Parking area	Yes
	Airport, aerodrome	Yes
	Public transport station/facilities	Yes
**Broad location type**	**Specific location type**	**Public place**
	Railway (other than station)	Yes
	Airstrip, helipad	Yes
	Bus stop, tax rank	Yes

© 2024 Too LS et al. *JAMA Network Open.*

	Loading bay, dock	Yes
	Other specified transport area: other	Yes
	Unspecified transport area: other	Yes
Recreational area, cultural area, or public building	Public playground	Yes
	Amusement park, theme park	Yes
	Public park	Yes
	Public building, non-cultural and non-religious	Yes
	Holiday park, campground	Yes
	Public religious place	Yes
	Holiday resort, retreat	Yes
	Building for recreational purposes, public and commercial	Yes
	Public toilet	Yes
	Cemetery, crematorium	Yes
	Off road park	Yes
	Showground, fairground	Yes
	Public building, cultural or religious	Yes
	Private building, clubhouse	Yes
	Other specified recreational, cultural area, or public building	Yes
	Unspecified recreational, cultural area, or public building	Yes
Commercial area	Shop, store	Yes
	Commercial garage	No
	Office building, office workplace	No
	Hospitality venue	Yes
	Motel, hotel	Yes
	Shopping centre, mall, arcade	Yes
	Market	Yes
	Storage facility	Yes
	Other specified commercial area	Case-by-case and manually coded
	Unspecified commercial area	Case-by-case and manually coded
Other place of occurrence	Wharf, pier, jetty	Yes
	Bridge, overpass	Yes
	Aircraft	Yes
	Watercraft	Yes
	Land vehicle	Yes
	Boat ramp	Yes
	Other specified place of occurrence	Yes
Medical service area	Hospital	Case-by-case and manually coded
	Outpatient clinic, health centre	Yes
	Health professional's office	Yes
	Nursing home	No
	Hospice, palliative care facility	No
	Rehabilitation centre	No
**Broad location type**	**Specific location type**	**Public place**
	Ambulance	No
	Ambulance depot, base	No
	Respite facility	No

© 2024 Too LS et al. *JAMA Network Open.*

	Other specified medical service area	Case-by-case and manually coded
	Unspecified medical service area	Case-by-case and manually coded
Residential institution area	Home for the elderly, retirement village	No
	Prison, youth training or detention centre	No
	Shelter, refuge, halfway house or crisis accommodation	No
	Military institution	No
	Police station	No
	Residential care facility	No
	Other specified residential institution area	No
	Unspecified residential institution area	No
Farm/other place of primary production	Area for growing of crops, market gardening, horticulture	No
	Area for raising or care of animals	No
	Area for growing of crops combined with raising/care of animals	No
	Timber plantation	No
	Paddock, field	No
	Hobby farm, property	No
	Other specified farm	No
	Unspecified farm	No
Industrial/construction area	Construction site	No
	Demolition site, derelict site	No
	Factory, plant, warehouse	No
	Mine and quarry	No
	Oil or gas extraction facility	No
	Shipyard	No
	Power station	No
	Garbage dump, refuse, scrap metal yard	No
	Abattoir	No
	Disused industrial or construction site	No
	Research laboratory	No
	Shearing shed	No
	Grain silo, grain storage facility	No
	Stockyards, sales yard	No
	Timber yard	No
	Transport depot, yard	No
	Tower - communication, power, radio, water	No
	Other specified industrial or construction area	No
**Broad location type**	**Specific location type**	**Public place**
	Unspecified industrial or construction area	No
Sports and athletics area	Sporting grounds (outdoor)	Yes

© 2024 Too LS et al. *JAMA Network Open.*

	Sporting area, hall (indoor)	Yes
	Public swimming centre	Yes
	Racetrack, racecourse	Yes
	Equestrian facility	Yes
	Skating rink, ice palace	Yes
	Skiing or snowboarding area	Yes
	Aero sport landing area	Yes
	Firearm range	Yes
	Recreational trail, track	Yes
	Other specified sports and athletics area	Yes
	Unspecified sports and athletics area	Yes
School, educational area	School, university, adult education institution	Yes
	Day care, kindergarten, play centre	Yes
	Sports and athletics area at school, educational area	Yes
	Playground at school, educational area	Yes
	Student accommodation	No
	Other specified school, educational area	Case-by-case and manually coded
	Unspecified school, educational area	Case-by-case and manually coded
Unknown	Unknown	No

© 2024 Too LS et al. *JAMA Network Open.*
