# Supplementary material for: High-Risk Suicide Locations in Australia
Source: JAMA Netw Open. 2024 Jun 20;7(6):e2417770. doi: 10.1001/jamanetworkopen.2024.17770 (PMC11190791; doi:10.1001/jamanetworkopen.2024.17770)
Supplement: Supplement 2. — Data Sharing Statement [file jamanetwopen-e2417770-s002.pdf]

Data Sharing Statement

Too. High-Risk Suicide Locations in Australia. *JAMA Netw Open*. Published June 20, 2024.
doi:10.1001/jamanetworkopen.2024.17770

Data
Data available: No

Additional Information
Explanation for why data not available: Data used in this study is confidential and are restricted to authors who are granted the data access for this study by the Victorian Justice Human Research Ethics Committee.
